# Protocol for separation of fungal extracellular vesicles using ultracentrifugation from solid medium cultures

**DOI:** 10.1016/j.xpro.2024.103069

**Published:** 2024-05-20

**Authors:** Alicia C. Piffer, Flavia C.G. Reis, Frédérique Moyrand, Bruna Montalvão, Marcio L. Rodrigues, Guilhem Janbon, Juliana Rizzo

**Affiliations:** 1 Institut Pasteur, Université Paris Cité, Unité Biologie des ARN des Pathogènes Fongiques, Département de Mycologie, 75015 Paris, France; 2 Instituto Carlos Chagas, Fundação Oswaldo Cruz (Fiocruz), Curitiba 81350-010, Brazil; 3 Centro de Desenvolvimento Tecnológico em Saúde (CDTS), Fiocruz, Rio de Janeiro 21040-361, Brazil; 4 Instituto de Microbiologia Paulo de Góes (IMPG), Universidade Federal do Rio de Janeiro (UFRJ), Rio de Janeiro 21941-902, Brazil; 5 Instituto de Biofísica Carlos Chagas Filho (IBCCF), Universidade Federal do Rio de Janeiro (UFRJ), Rio de Janeiro 21941-904, Brazil

**Keywords:** Cell Biology, Cell isolation, Cell-based Assays, Flow Cytometry, Microbiology, Model Organisms

## Abstract

Extracellular vesicles (EVs) have been identified in diverse fungi, including human pathogens. In this protocol, we present two techniques for isolating and analyzing fungal EVs. The first is for high-throughput screening, and the second is for yielding concentrated samples suitable for centrifugation-based density gradients. We describe steps for analytical assays such as nano-flow cytometry and nanoparticle tracking analysis to measure EV dimensions and concentration. EV suspensions can serve diverse assays, including electron microscopy, compositional determination, and cell-to-cell communication assays.

For complete details on the use and execution of this protocol, please refer to Rizzo et al.,^1^ Rizzo et al.,^2^ Reis et al.,^3^ and Reis et al.^4^

## Highlights

• Steps for fungal EV isolation for screenings
• Facilitated approach for concentrated fungal EV samples
• EV enrichment steps using an iodixanol gradient
• Steps for the quantification of single-particle concentration and size

## Abstract (editor highlights)

Publisher’s note: Undertaking any experimental protocol requires adherence to local institutional guidelines for laboratory safety and ethics.

## Graphical abstract

**Figure:**
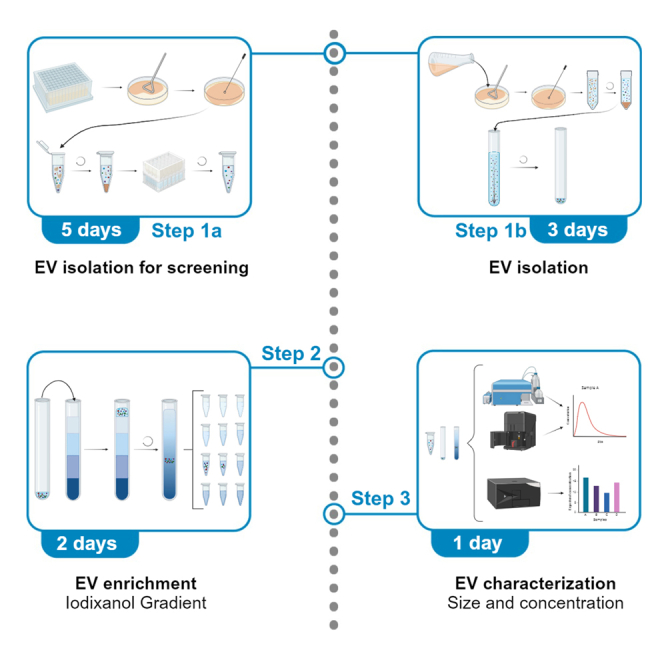

## Before you begin

According to the latest Minimal information for studies of extracellular vesicles (MISEV 2023), extracellular vesicles (EVs) refer to particles that are released from cells, are delimited by a lipid bilayer, and cannot replicate on their own.^5^ Fungal EVs enclose diverse virulence factors, immunogens, and molecules with unknown functions, mediating biofilm formation, contributing to antifungal resistance, and stimulating host immune responses.^6^ To better understand the fungal EV biosynthetic processes, their biomarkers, and multiple biological roles, the protocol for EV isolation from solid culture media has proven to be a suitable method. The techniques outlined below have been designed to separate, enrich, and characterize the biophysical and biochemical properties of EVs originating from the fungus *Cryptococcus neoformans*. It is worth noting that this protocol can be adapted to isolate EVs from other fungal species. To achieve this, carefully considering the appropriate growth condition is essential, tailored to the specific organism being studied.

### Preparation of medium and buffer

**Timing: 1 day**

1. Prepare all the media and buffers necessary for the procedure. Check the materials and equipment section.
  a. Yeast extract-peptone-dextrose (YPD)-liquid.
  b. YPD-agar.
  c. Synthetic dextrose (SD)-agar.
  d. Phosphate Buffered Saline (PBS).

### Preparation of iodixanol gradient

**Timing: 2 h**

2. Prepare the iodixanol 50% solution as described in the materials and equipment section.
3. Prepare the different concentrations of iodixanol (40%–5%) following the Table 1.

**Table 1:** Specifications to prepare the different solutions of Iodixanol and the Iodixanol Gradient

Gradient solution	Iodixanol 50%	Gradient buffer	Volume added in the ultra-clear tube
40%	5.6 mL	1.4 mL	3 mL
20%	2.8 mL	4.2 mL	3 mL
10%	1.4 mL	5.6 mL	3 mL
5%	0.7 mL	6.3 mL	2.5 mL

Final volume for each solution is 7 mL.

***Note:*** Routinely, we prepare 7 mL of each concentration of iodixanol, which is the volume necessary to prepare two gradient tubes. The final volume could be changed regarding the number of gradient tubes needed.

**CRITICAL:** Remind to always balance the centrifuge. As iodixanol is heavier than water, is necessary to run two gradient tubes at the same time.

4. Process to assemble the gradient (volumes of each solution also described in Table 1).
  a. Add 3 mL iodixanol solution 40% to an Ultra-clear tube. Freeze the tube in −80°C for 20 min.
  b. Remove the tube from the −80°C freezer and add 3 mL iodixanol solution 20%. Freeze the tube in −80°C for 20 min.
  c. Remove the tube from the −80°C freezer and add 3 mL iodixanol solution 10%. Freeze the tube in −80°C for 20 min.
  d. Remove the tube from the −80°C freezer and add 2.5 mL iodixanol solution 5%. Freeze the tube in −80°C for 20 min.

***Note:*** It is possible to conduct the density gradient without an intervening freezing step between the different concentrations of iodixanol. Nevertheless, the procedure requires careful execution due to the possibility of unintentionally mixing fractions during the process.

5. Store the gradient at −20°C.

**Pause point:** The gradient can be stored for up to 1 week at −20°C.

## Key resources table

**Table:** 

REAGENT or RESOURCE	SOURCE	IDENTIFIER
**Chemicals, peptides, and recombinant proteins**		

Yeast nitrogen base (YNB) without amino acids	BD Difco	291940
OptiPrep density gradient medium (iodixanol)	Sigma-Aldrich	D1556
TE buffer pH 8.0	Invitrogen	AM9849

**Critical commercial assays**		

Amplex red cholesterol assay kit	Invitrogen	A12216

**Experimental models: Organisms/strains**		

*Cryptococcus neoformans KN99α* strain	Nielsen et al., 2003.^7^	N/A

**Software and algorithms**		

NF Profession 2.0	NanoFCM Co. Ltd.	N/A
NTA 3.0 software	Malvern Panalytical	N/A

**Other**		

BREATHseal sealer	Greiner Bio-One	676051
Inoculation spreader	Sarstedt AG & Co	86.1569.005
Inoculation loops	Sarstedt AG & Co	86.1562.010
96-well filter plate porosity 0.45 μm	Millipore	MSHAS4510
96-well plate flat bottom	TPP	92096
50 mL polypropylene bottle for centrifugation	Beckman Coulter, Inc.	357003
Vacuum filtration 0.22 μm PES membrane filter	Thermo Scientific	566-0020
Syringe filter porosity 0.45 μm	ClearLine	051797
Syringe filter porosity 0.02 μm	Whatman	6809-2102
Ultra-clear centrifuge tubes	Beckman Coulter, Inc.	344059
26.3 mL polycarbonate bottle	Beckman Coulter, Inc.	355618
Centrifuge	Eppendorf	MiniSpin 5452000010
Centrifuge	Eppendorf	5810 R
Centrifuge	Beckman Coulter, Inc.	Avanti J-25
Ultracentrifuge	Beckman Coulter, Inc.	Optima XPN-80
0.6 mL Maxymum Recovery microtubes	Axygen, Inc	MCT-060-L-C
NanoFCM quality control nanospheres (QC beads), 250 ± 5 nm, 2E+10 particles/mL	NanoFCM Co. Ltd.	QS2503
Silica nanospheres cocktail (size beads), 68–155 nm, 100×	NanoFCM Co. Ltd.	S16M-Exo
NanoFCM cleaning solution, 50×	NanoFCM Co. Ltd.	N/A
NanoFCM Flow NanoAnalyzer	NanoFCM Co. Ltd.	N/A
NanoSight	Malvern Panalytical	N/A
96-well microplate, half area, black	Greiner Bio-One	675077
Plate reader	Tecan Trading AG	Infinite M Plex
Extran MA 02	Merck	107553
ISOTON II diluent	Beckman Coulter, Inc.	8448011
Particle counter	Beckman Coulter, Inc.	Z1 Coulter

## Materials and equipment

**Table:** YPD liquid

Reagent	Final concentration	Amount
Glucose	2%	20 g
Bacto peptone	2%	20 g
Bacto yeast extract	1%	10 g
MilliQ H_2_O	N/A	1000 mL
**Total**	**N/A**	**1000 mL**

Sterilize by autoclavation. Store at 20°C–25°C.

**Table:** YPD-agar

Reagent	Final concentration	Amount
Glucose	2%	20 g
Bacto peptone	2%	20 g
Bacto yeast extract	1%	10 g
Bacto agar	2%	20 g
MilliQ H_2_O	N/A	1000 mL
**Total**	**N/A**	**1000 mL**

Sterilize by autoclavation. Use 25 mL per plate and store at 4°C for up to one week.

**Table:** SD medium 10x

Reagent	Final concentration	Amount
Glucose	20%	20 g
YNB w/o amino acids	10x	6.7 g
MilliQ H_2_O	N/A	100 mL
**Total**	**N/A**	**100 mL**

Sterilize by filtration in 0.22 μm. Store at 20°C–25°C.

**Table:** Bacto-Agar solution 2.2%

Reagent	Final concentration	Amount
Bacto agar	2.2%	20 g
MilliQ H_2_O	N/A	900 mL
**Total**	**N/A**	**900 mL**

Sterilize by autoclavation. Store at 20°C–25°C.

• **Medium SD-agar:** heat the bacto-agar solution until it is liquid again. When the temperature reaches 50°C–55°C add 100 mL of SD medium 10x in 900 mL bacto-agar solution. Mix well and add 25 mL to each petri dish.

Store at 4°C for up to one week.

**Table:** Concentrated (10x) PBS

Reagent	Final concentration	Amount
KH_2_PO_4_	19.1 mM	1.3 g
K_2_HPO_4_	71.7 mM	6.25 g
NaCl	1.49 M	43.55 g
MilliQ H_2_O	N/A	500 mL
**Total**	**N/A**	**500 mL**

Sterile filter through a 0.22 μm filter system. Store at 20°C–25°C.

• **PBS 1x:** add 10 mL of PBS 10x in 90 mL H_2_O MilliQ. Sterile filter through a 0.22 μm filter system. Store at 20°C–25°C.

**Table:** Gradient Buffer

Reagent	Final concentration	Amount
Tris HCl pH7.5 1 M	10 mM	0.5 mL
Sucrose 2 M	0.25 M	6.25 mL
H_2_O MilliQ	N/A	43.25 mL
**Total**	**N/A**	**50 mL**

Sterile filter through a 0.22 μm filter system. Store at 20°C–25°C.

• **Iodixanol 50%:** add 10 mL of Iodixanol 60% (OptiPrep Density Gradient Medium) in 2 mL of gradient buffer. The final volume is 12 mL. Store at 20°C–25°C.

## Step-by-step method details

### EV isolation for screening (step 1a)

**Timing: 3–5 days**

**Figure 1:**
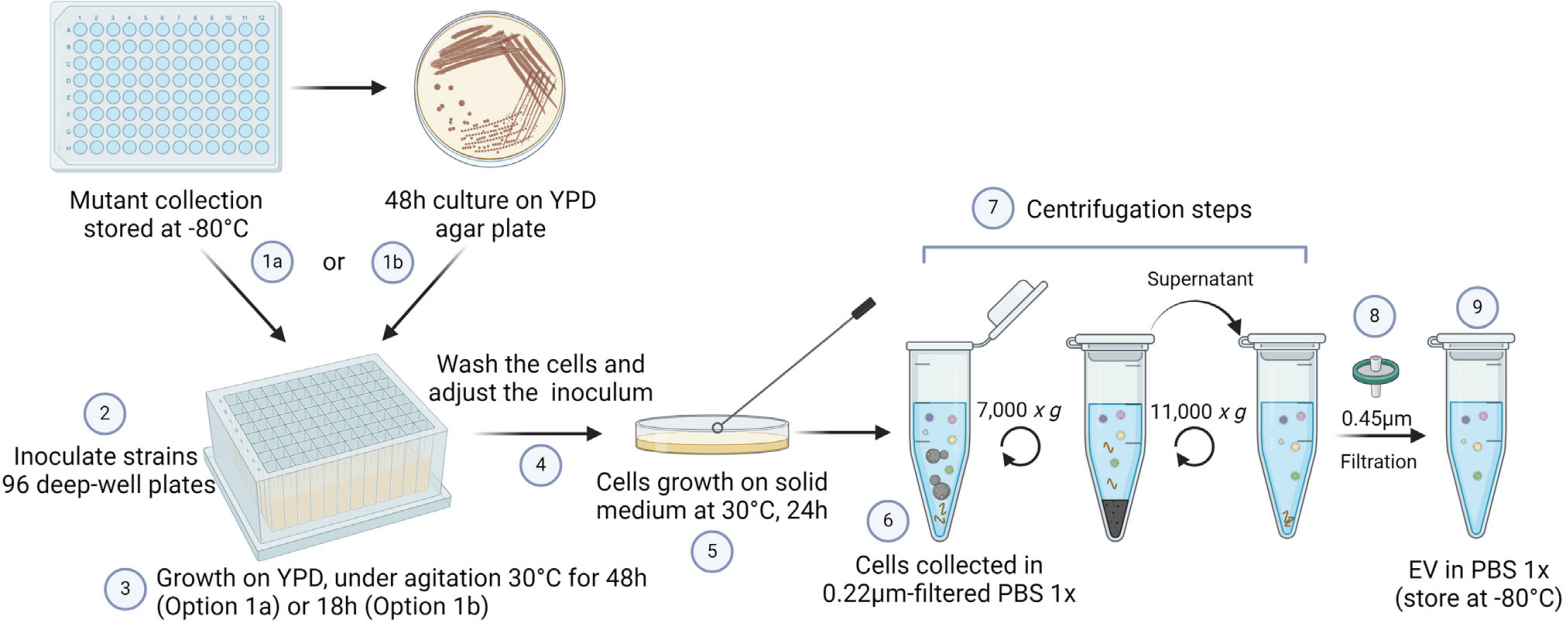
Graphical illustration of Step 1a Cell culture steps (1–5) and EV isolation steps (6–9) for high-throughput screening of EV production.

This protocol describes the methodology to obtain EVs on a high-throughput scale. The primary objective is to expedite the comparative analysis of various strains, serving as knockout mutants sourced from a collection. Within our laboratory, the strains are stored at −80°C in a 40% glycerol solution. Of note, the protocol might be applicable for testing the effects of potential inhibitors of EV formation as well. A graphical illustration of Step 1a is depicted in Figure 1.

1. Pre-inoculum of yeast cells.
  a. In a 96 deep-well plate add 1 mL of liquid YPD in each well.
  b. Add a loop of cells from a 48 h culture on YPD agar plate, previously obtained from the stock at −80°C.
  c. Seal the plates with breathseal sealer and incubate for 18 h at 30°C under agitation.

***Optional:*** The pre-inoculum step can also be started by adding 20 μL of the cell stock in 1 mL of liquid YPD and incubating it for 48 hours at 30°C under agitation.

2. Plating in SD-agar plates.
  a. Wash the cells twice with sterile H_2_O.
    i. Centrifuge the deep-well plate at 3000 × *g* for 10 min.
    ii. Remove the supernatant with a multichannel pipette.
    iii. Add 1 mL of sterile H_2_O and pipette up and down approximately 10 times to wash the cells.
    iv. Repeat the steps i-iii twice.
  b. Determine the OD_600_ of the cell suspensions (200 μL) using a microplate reader.
  c. Adjust the cells to OD_600_ = 0.4.
    ***Note:*** The OD_600_ value was determined for *C. neoformans*. For other species is necessary to find the adequate OD_600_ corresponding to 3.5 × 10^7^ cells/mL.
  d. Spread 300 μL of the suspension onto SD-agar plates using a T or L-shaped cell spreader.
    Incubate the plates for 24 h at 30°C.
    **CRITICAL:** Let the drops of water usually formed during the preparation of SD-agar plates dry completely before spreading out the cells.
3. Collecting the cells and isolating the EVs.
  a. Gently recover the cells from the agar plates with an inoculation loop (Methods video S1) in a sterile 2 mL tube containing 1 mL of 0.22 μm-filtered PBS 1x.
    ***Note:*** Keep the tubes with the cells on ice when collecting all the conditions or strains.
    Methods video S1. Collecting cells and EVs from SD-agar plates, related to Steps 1a and 1b
  b. Suspend the cells by gently pipetting up and down.
    **CRITICAL:** Do not vortex the cells.
  c. Collect an aliquot of the cells to count the total number of cells collected considering the final volume reached for each condition.
    ***Optional:*** The recovered cells can be fixed with 4% paraformaldehyde to be counted later on.
  d. Centrifuge the tubes at 7,000 × *g* for 5 min.
  e. Carefully pipet the supernatant into a new 1.5 mL sterile tube without touching the pellet.
  f. Centrifuge the tubes at 11,000 × *g* for 5 min.
  g. Transfer the supernatant to a 96-well filter plate with 0.45-μm pore size and centrifuge at 1,200 × *g* for 3 min at 4°C.
  h. Collect the passed-through supernatant containing EVs and store it at −80°C for further evaluation.

### EVs isolation (step 1b)

**Timing: 3 days**

**Figure 2:**
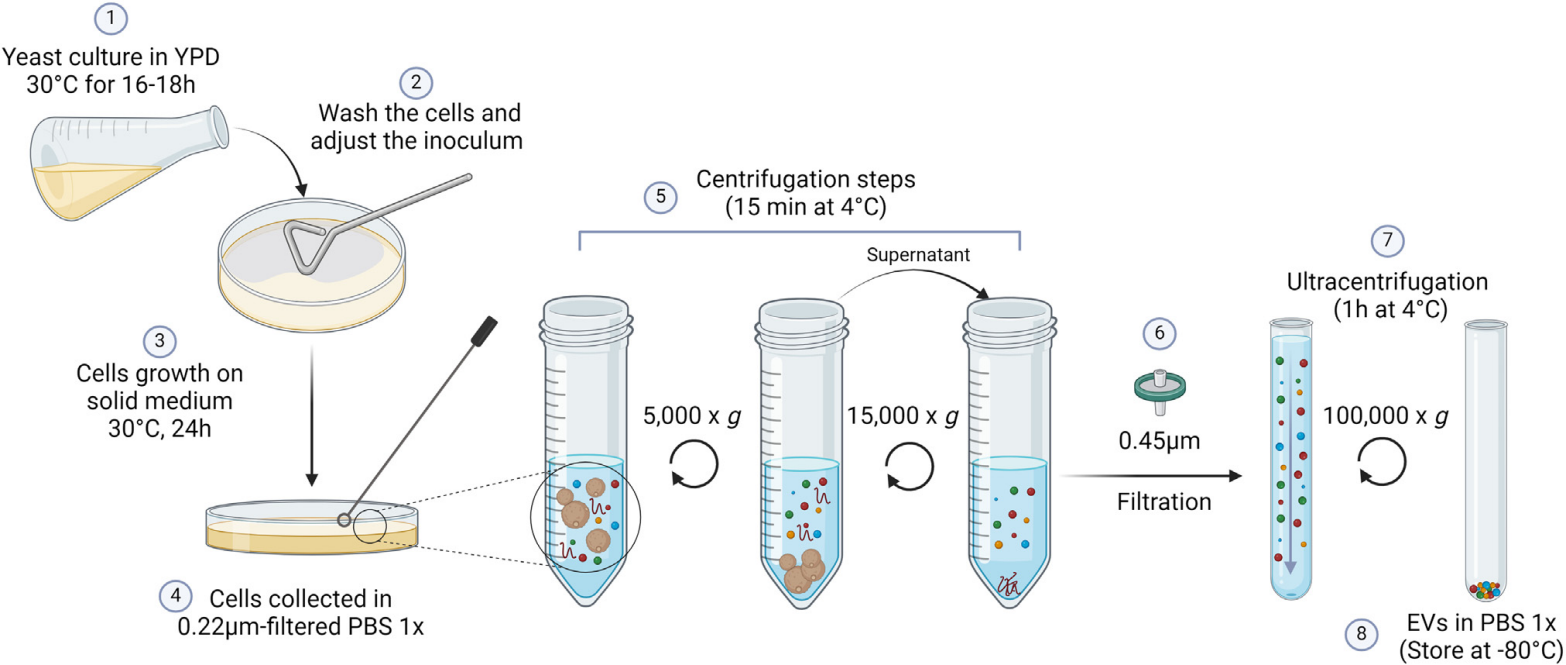
Graphical illustration of Step 1b Cell culture steps (1–3) and EV isolation steps (4–8) for facilitated approach yielding concentrated EV samples.

This protocol describes the process of obtaining concentrated EV samples. Cells are usually stored at −80°C in a 40% glycerol solution and strains of interest are streaked from −80°C every week on YPD-agar plates. A graphical illustration of Step 1b is depicted in Figure 2.

4. Pre-inoculum of yeast cells.
  a. Add 10 mL of liquid YPD in a 50 mL Erlenmeyer flask.
  b. Add a loop of cells grown previously on the YPD plate for 48 h at 30°C.
    i. Incubate for 16–18 h at 30°C under agitation.
5. Plating on SD-agar.
  a. Wash the cells twice with sterile H_2_O.
    i. Collect 2 mL of the cell suspension in a 2 mL tube.
    ii. Centrifuge at 11,000 × *g* for 3 min.
    iii. Remove the supernatant.
    iv. Add 1 mL of sterile H_2_O.
    v. Repeat the steps ii-iv twice.
  b. Count the cells.
    ***Note:*** Generally, we use a Z1 Coulter Particle Counter machine (Beckman Coulter) for determination of particle numbers. This process usually follows a 100x sample dilution and the addition of 100 μL of this suspension to 10 mL of Isoton II Diluent. From this last dilution, the equipment uses 500 μL to count the cells. To obtain the density of cells/mL, the number provided by the equipment is multiplied by 2 × 10^4^. The cells can be also counted by other approaches as by Thoma or Neubauer cell counting chambers and different calculations will be necessary to determine the number of cells per mL.
  c. Dilute the cells to obtain a suspension of 3.5 × 10^7^ cells/mL in sterile H_2_O.
  d. Distribute 300 μL of the suspension on SD-agar plates using a T or L-shaped cell spreader.
  e. Incubate the plates for 24 h at 30°C.
    **CRITICAL:** Let SD-agar plates dry completely before spreading out the cells.
    ***Note:*** Typically, 6 SD-agar plates provide adequate EVs for executing the subsequent procedures; nevertheless, this quantity should be adjusted based on the fungus or the strain under consideration as well as the posterior analyses. For proceeding with the iodixanol gradient (Step 2), we usually use 12 SD-agar plates.
6. Collect the cells and obtain the EVs.
  a. Collect the cells with a loop in a sterile Polypropylene Bottle for centrifugation containing 10 mL of 0.22 μm-filtered PBS 1x.
    ***Note:*** Collect a maximum of 10 plates in 10 mL of 0.22 μm-filtered PBS 1x. If you plan to collect more plates, divide them into 2 tubes.
  b. Suspend the cells by gently pipetting (do not vortex the cells).
    i. With the pipette, determine the total volume of cell suspension.
    ii. Collect an aliquot of the cells and determine the concentration of the cells.
      ***Optional:*** The recovered cells can be fixed with 4% paraformaldehyde to be counted later on.
  c. Centrifuge the tubes at 5,000 × *g* for 15 min at 4°C.
  d. Carefully pipet the supernatant into a new sterile Polypropylene Bottle for centrifugation without touching the pellet of cells.
  e. Centrifuge the tubes at 15,000 × *g* for 15 min at 4°C.
  f. Filter the remaining supernatant in a sterile 0.45 μm filter with a syringe in a tube specific for ultracentrifugation.
    ***Note:*** In the laboratory, we use either the SW41 Ti rotor or the fixed-angle rotor type 70 Ti. For the SW41 Ti rotor we use the Ultra-clear tube and for the rotor 70 Ti we use Polycarbonate tubes. To reuse the tubes, it is important to clean them according to the manufacturer’s guide.
    ***Note:*** If tubes are meant for reuse, cleaning and sterilization processes are crucial. The cleaning procedure involves manual washing with Extran detergent (20%), followed by extensive rinsing with milliQ-water to eliminate any lingering detergent residue. Subsequently, the tubes are left to air dry. Prior to usage, the tubes are rinsed with 70% ethanol and allowed to air dry under a biosafety cabinet. Right before adding the sample, three additional washes are conducted using 0.22 μm-filtered PBS. Tubes that have been used should always be inspected for signs of any deformation or cracking.
  g. Centrifuge at 100,000 × *g* for 1 h at 4°C, rotor acceleration and deceleration on maximum.
    **CRITICAL:** It is essential to accurately balance the tubes for the ultracentrifugation. An unbalanced rotor may cause equipment damage and personal injury.
  h. Discard the supernatant by inverting the tube. The EV pellet must be in the bottom of the tube or at the edge of the tube, for swing or fixed-angled rotor, respectively. Invert the tube in an absorbent paper for some seconds to remove the remaining supernatant.
    **CRITICAL:** Work as fast as possible to prevent the EV pellet from dissociating and drying.
  i. Suspend the EV pellet in up to 300 μL of 0.22 μm-filtered PBS 1x by gently pipetting 10–15 L times and rinsing the edges of the tube.
  j. Aliquot the EVs in several sterile tubes and store them at −80°C.
    ***Note:*** It is important to store the EVs in multiple aliquots at −80°C to prevent the need for repetitive freezing and thawing of the samples.

### EV enrichment using an iodixanol gradient (step 2)

**Timing: 2 days**

This protocol describes how to enrich the samples on EV content using a density gradient.

**CRITICAL:** During all protocol steps, move the tube slowly to not mix the fractions.

7. Thaw the gradient at 4°C.

***Note:*** it takes at least 3 hours to thaw completely the gradient. Do not use it if is not completely thawed.

8. Add the EV sample (obtained following Step 1b) slowly on the top of the gradient.

***Note:*** To recover a substantial amount following gradient elution, suspend the EVs obtained in Step 1b in 500 μL, set aside an aliquot of 20 μL for quantification analysis, and add the remaining volume on the top of the gradient.

9. Centrifuge at 100,000 × *g* for 18 h at 4°C using the SW41 Ti rotor. Ultracentrifugation acceleration on maximum and deceleration on 7.
10. Collect the 12 fractions (F1-F12) pipetting slowly 1 mL for each fraction from the top to the bottom of the gradient and add each of them in new Ultra-clear tubes.

***Note:*** It is important to gently handle the tube and slowly pipette the fractions in order to avoid mixing gradient fractions during collection.

11. Resuspend each fraction in 9 mL of 0.22 μm-filtered PBS 1x.
12. Centrifuge all the tubes at 100,000 × *g* during 1 h at 4°C using the SW41 Ti rotor.

***Note:*** Due to the limitation of using only 6 tubes per centrifugation in the SW41 Ti rotor ultracentrifuge, we keep the additional tubes on ice or in the refrigerator throughout the ultracentrifugation process.

13. Discard the supernatant by inverting the tube and dry it in an absorbent paper for some seconds to remove the remaining supernatant.
14. Suspend the pellet from each fraction in 100 μL of 0.22 μm-filtered PBS 1x by pipetting several times and rinsing the edges of the tube.
15. Transfer to sterile tubes and store at −80°C for future analysis.

### Determining the size and concentration of the EVs (step 3)

**Timing: 1 day**

This protocol describes the quantification of single-particle concentration and size. It is possible to characterize the samples obtained by Steps 1a, 1b, and 2 by employing the complementary methodologies outlined subsequently. The NanoFCM Flow NanoAnalyzer (NanoFCM Co. Ltd.) facilitates the quantification of nanoparticles within the size range of 40–1000 nm. The NanoSight (Malvern Panalytical Ltd.) works within the particle size range of 10–1000 nm.

Part 1: NanoFCM.

16. Prepare cleaning solutions and dilute standard beads.
  ***Note:*** Make sure to use microcentrifuge tubes that are compatible with the nanoFCM acquisition
  a. Cleaning solution: Add 150 μL of NanoFCM cleaning solution 1x in a 0.6 mL microcentrifuge tube.
  b. NaOH 1 N solution: Add 150 μL NaOH 1 N solution (pre-filtered in a 0.02 μm filter) in a 0.6 mL microcentrifuge tube.
  c. NanoFCM QC beads (250 nm SiNPs): Add 1 μL QC Beads in 99 μL MilliQ H_2_O (1:100 dilution) in a 0.6 mL microcentrifuge tube. This solution will be used as a concentration standard.
  d. 68–155 S16M-Exo sizing beads (SiNPs cocktail): Add 1 μL cocktail beads in 99 μL MilliQ H_2_O (1:100 dilution) in a 0.6 mL microcentrifuge tube. This solution will be used as a size standard.
  e. MilliQ water: Add 150 μL of 0.02 μm-filtered- MilliQ H_2_O in a 0.6 mL microcentrifuge tube.
    **CRITICAL:** QC beads and 68–155 S16M-Exo sizing beads solutions must be diluted daily.
17. Start up the machine following the manufacturer’s instructions.
18. Align the machine and calibrate concentration using QC beads and size 68–155 S16M-Exo sizing beads.
  a. Click Manual Operation located underneath the CCD camera and select single-photon counting modules (SPCM) to switch on detectors.
  b. Select 250 nm Std FL SiNPs from Samp. Inf. dropdown.
  c. Load QC beads into the loading bay. Select Boosting from Sample Flow and boost it for 90 s to introduce beads into the system.
  d. Select Sampling from Sample Flow dropdown. Select the Auto sampling button (green light) to keep the pressure stable throughout use, which usually is around 1.0 kPa.
  e. Click on Auto-threshold and select large signal.
  f. Align the equipment using the laser control panel.

**Figure:**
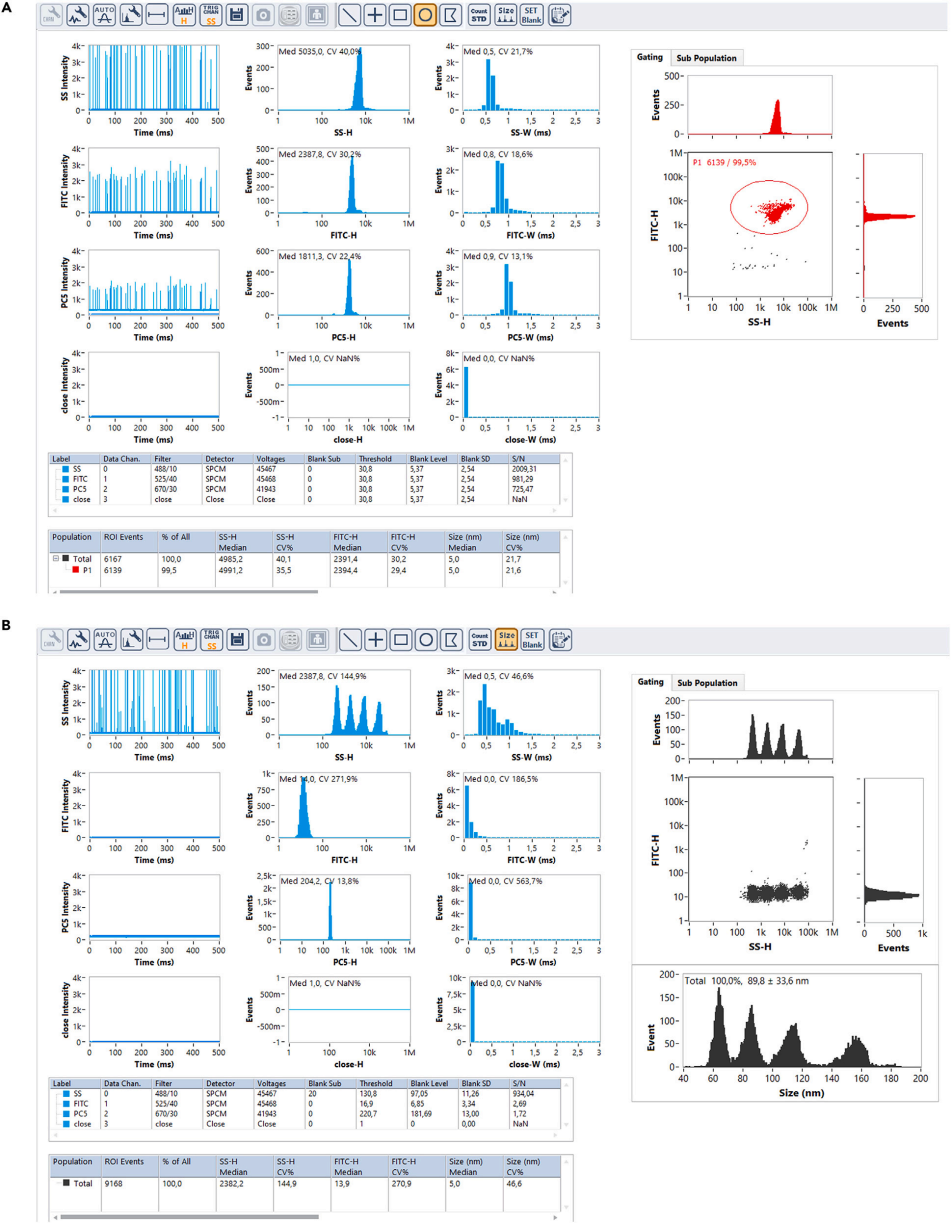
    Methods S1: NanoFCM data analysis, related to Step 3. (A) Concentration standard setting using QC beads (250 nm SiNPs) showing the histograms with uniform and narrow peaks expected in a good alignment. (B) Size standard setting using 68–155 S16M-Exo sizing beads (SiNPs cocktail) showing the four expected peaks related to the four different bead sizes.
    **CRITICAL:** When achieving optimal alignment, it is crucial to verify that the histograms exhibit uniform and narrow peaks. If not, make manual adjustments to the horizontal and vertical positioning of the optical lenses, or optimize the alignment of the laser focusing lens by adjusting the laser knobs.
  g. Once you have aligned the machine and the QC beads are still being sampled select Time to Record on the Acquisition Controls panel. When finished a popup will appear to save the file.
  h. Select Unload from Sample Flow. Remove the tube and load cleaning solution tube. Select Boosting from Sample Flow dropdown and boost it for 90 s.
  i. Select Unload from Sample Flow dropdown and rinse the sample line with milliQ H_2_O.
  j. To calibrate the size beads: Select 68–155 nm S16M-Exo from Samp. Inf. dropdown.
  k. Load S16M-Exo beads into loading bay. Select Boosting from Sample Flow dropdown and boost it for 90 s.
  l. Select Sampling from Sample Flow drop down.
  m. Click on Auto-threshold and select small signal, and click on SPCM correction button to change SS sub value to 20.
  n. Select Time to Record on the Acquisition Controls panel. When finished a popup will appear to save the file.
  o. Select Unload from Sample Flow dropdown. Remove the tube and repeat steps h and i to clean the equipment. The system is now ready to run the blank and samples.
19. Recording blank and samples.
  ***Note:*** Dilute the samples in TE buffer (10 mM Tris, 1 mM EDTA pH 8) filtered in 0.02 μm filter. Pipette-mix only, do not vortex the sample because it could lead to microbubble formation.
  a. Load the TE buffer tube (previously filtered in 0.02 μm filter) into the loading bay. This record is needed to remove the background in the analysis after.
  b. Select Boosting from Sample Flow dropdown. Allow it to boost for 90 s.
  c. Select Sampling from Sample Flow dropdown. After that the sample signal appears in the graphs select Time to Record.
  d. When finished a popup will appear to save the file.
  e. Select Unload from Sample Flow dropdown.
  f. Remove the tube and load the cleaning solution 1x tube. Select Boosting from Sample Flow dropdown and boost it for 90 s.
  g. Select Unload from Sample Flow dropdown and rinse the capillary with milliQ H_2_O.
  h. Load the sample tube (diluted) and repeat the procedure b-g with all the samples.
    **CRITICAL:** The number of events should be between 4000 and 13000. If the sample presents a number of events outside the range, it is necessary to redo the dilution.
20. After recording all the samples, proceed to the shutdown following the manufacturer’s instructions.
21. Data Analysis.
  a. Launch the NanoFCM software NF Profession 2.0 and load the folder containing the data to be analyzed.
  b. Select the QC beads file, click on the Autotreashold icon and select large signal.
  c. Select the QC beads with the circle gate icon and click on the count standard icon.
  d. A window will open when is possible to specify the concentration (2.2 × 10^10^ beads/mL) and the dilution factor (100). The concentration standard is now set.
    ***Note:*** This concentration may vary between batches.
  e. Select the 68–155 S16M-Exo2 size file.
  f. Click on the SPCM correction window and change SS sub value to 20. Click on the Autotreashold icon and select small signal. Click on SIZE MESF icon.
  g. A window will open: choose S16M-Exo in the Standard dropdown and click on Find peak. A regression line should appear. The size standard is now set.
  h. To remove the background, select the TE buffer file and change to 200 the scale in the graph SS intensity vs. time.
  i. Click on Autotreashold icon and select small signal. Click on set blank icon to remove the background.
  j. Select the sample files and specify the dilution factors.
  k. Click on sample analysis and export the data as a pdf file containing size and concentration information.

Part 2: NTA

***Note:*** NTA can be conducted using various equipment from different manufacturers. Our primary experience involves utilizing a Malvern Nanosight nanoparticle tracking analyzer (LM10 or NS300 models), equipped with a 488 or 642 nm lasers, a sCMOS camera, and a syringe pump (Malvern Panalytical, Malvern, United Kingdom).

**CRITICAL:** Prior to analysis, it is crucial to ensure the cleanliness of the system. This involves using a syringe filled with distilled water. The syringe is attached to the syringe pump, connected to the equipment's cannulas, and flushed with 1 mL of distilled water three times. This cleaning step is repeated between the analysis of each sample.

22. To optimize the detection range of the equipment, adjust the EV samples to a density ranging from 1 × 10^8^ to 1 × 10^9^ particles per milliliter in filter-sterilized (0.22 μm) PBS.
23. Introduce the EV samples into the system using a 1 mL syringe connected to the syringe pump and the cannulas. The samples are injected with a continuous flow at a preset speed, usually around 50.
24. Configure the software settings for optimal NTA analysis. Calibrate the software to record three videos (each lasting 60 s). Adjust the camera level within the range of 10–15, and set the fluid viscosity to match that of water.

***Note:*** During the analysis process, the sample is injected through the equipment's chamber. As particles pass through the laser, their concentration and diameter are determined by the equipment's software (e.g., NTA 3.0 Software, Malvern Panalytical in our case), employing the Stokes-Einstein equation. The three recorded videos are subsequently analyzed with the same software.

25. Adjust the detection threshold to a range of 7–15 for better visualization of nanoparticles.
26. After completing the analysis of a sample, perform equipment cleaning to prevent contamination of the equipment’s chamber or cannulas by particles.

Part 3: Amplex Red Cholesterol Assay

This assay quantifies the amount of cholesterol in the sample by the following reactions: cholesterol esterase digests cholesteryl esters and releases cholesterol, which is then detected in the enzyme-coupled reaction with Amplex Red reagent. Fungal membranes do not contain cholesterol, but ergosterol which can be efficiently measured by the described reactions. Ergosterol is also present in fungal EV membranes,^8^ therefore, measuring ergosterol makes it possible to indirectly determine the EV content in the sample.

***Note:*** The following procedure is designed for use with a fluorescence multiwell plate reader with a final volume of 50 μL in a 96-well microplate, half area, black, flat bottom. To use a standard fluorometer, volumes must be increased accordingly.

27. Prepare the solutions following the manufacturer’s instructions.

***Note:*** Aliquot the working solution and stock it at −20°C. This solution turns pink when stocked for long time and reducing the freeze and thaw cycles will delay the color change.

28. Experimental Protocol.
  a. Prepare standard curves in separate wells of a 96 well-plate. Prepare serial two-fold dilutions (as described in Table 2) and mix by pipetting each well. The final volume in the well is 25 μL. Discard the 25 μL in excess in the well of 0.625 μM.

**Table 2:** Specifications to prepare cholesterol standard curve

Concentration μM	Well∗	Reaction buffer 1X	Cholesterol solution
40 μM	A1		50 μL stock solution 40 μM
20 μM	A2	25 μL	25 μL of well A1
10 μM	A3	25 μL	25 μL of well A2
5 μM	A4	25 μL	25 μL of well A3
2.5 μM	A5	25 μL	25 μL of well A4
1.25 μM	A6	25 μL	25 μL of well A5
0.625 μM	A7	25 μL	25 μL of well A6
0	A8	25 μL	

∗Example. Can be done in other wells.
  b. Dilute the sterol-containing samples (EVs) in 1X Reaction Buffer directly in the microplate well. Mix by pipetting.
    ***Note:*** Typically, for EVs obtained according with Step 1a, samples that came from 1 plate are diluted 2.5 times (10 μL sample + 15 μL 1x Reaction Buffer).
  c. Start the reactions by adding 25 μL of the working solution to each microplate well with a multichannel pipet.
  d. Mix by gently pipetting.
  e. Remove air bubbles.
  f. Incubate the reactions for 30 min at 37°C, protecting from light by wrapping the microplate in aluminum foil.
  g. Measure the fluorescence in a fluorescence microplate reader (TECAN INFINITE 200 according with our experience) using excitation at 560 nm and emission detection at 590 nm.

## Expected outcomes

**Figure 3:**
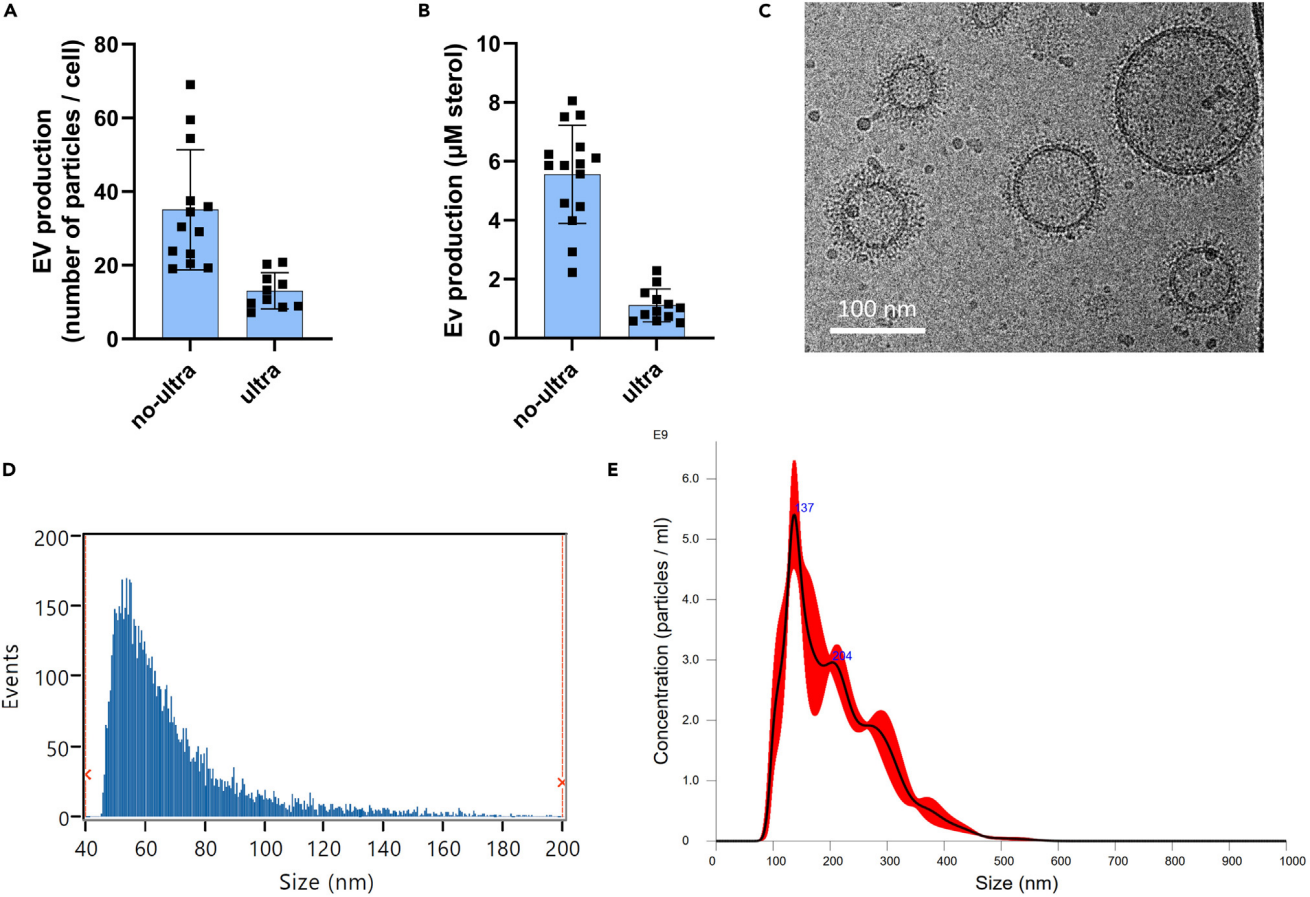
Characterization of EV derived from *C. neoformans* grown on SD solid medium (A) Analysis of EV production obtained by Step 1a protocol (no ultra) e Step 1b protocol (ultra) acquired by nanoFCM. (B) Total sterol content of EV isolated by Step 1a and 1b protocols normalized by 10^9^ cells. In order to compare the protocols, the EV suspensions of both protocols were considered in a final volume of 1 mL. Error bars show means ± SD. (C) Cryo-transmission electron microscopy (Cryo-EM) image of EVs obtained by Step 1b protocol. Scale bar = 100 nm. (D) EV size diameter distribution profiles by nanoFCM and (E) by Nanosight. Size profiles are derived from one representative EV sample.

The described protocols explored the EV isolation steps, followed by single-particle characterization from *C. neoformans*-derived particles growing on SD solid media. The expected number of cells collected after 24 h growth at 30°C is around 1.5 to 2.5 × 10^9^ cells/plate. Using the NanoAnalyzer, the EV isolation for screening protocol without ultracentrifugation (Step 1a) and the standard EV isolation by ultracentrifugation protocol (Step 1b) for KN99α strain are expected to yield EVs around 20–70 particles/cell and 5–20 particles/cell, respectively (Figure 3A).

For the sterol quantification using the Amplex Red Cholesterol Assay, one can expect to obtain 2 – 8 μM sterol/10^9^ cells (EV suspended in 1 mL) and 5–25 μM sterol/10^9^ cells (EV suspended in 100 μL), following protocols Step 1a and Step 1b, respectively (Figure 3B). Examples of isolated EVs from Step 1b protocol by Cryo-electron microscopy (Cryo-EM) are provided evidencing rounded shape particles delineated by lipid bilayers (Figure 3C). For some fungi, EVs were described to be decorated by a fibrillar material.^1^ For detailed information on EV sample preparation for Cryo-EM, please refer to Rizzo et al., 2021^1^ and Zarnowski et al., 2022.^9^

**Figure 4:**
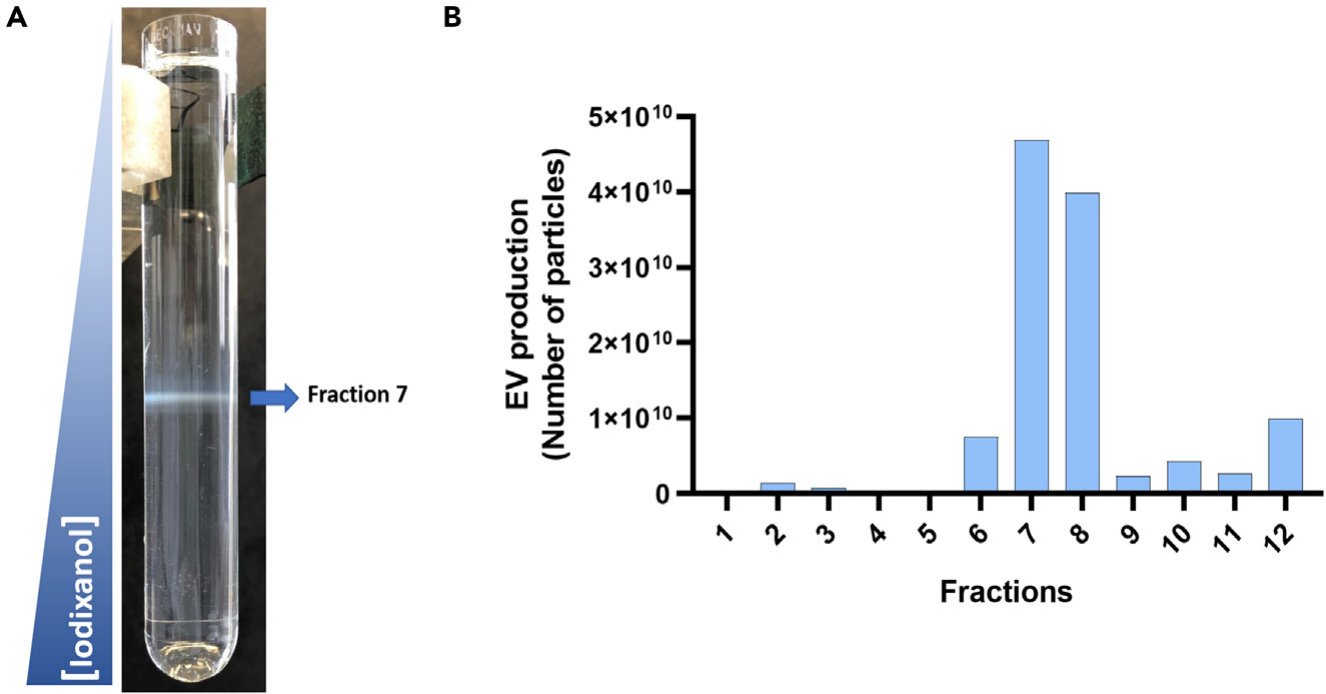
Characterization of *C. neoformans* EVs fractions after a step of enrichment using density gradient (Step 2) (A) Picture of iodixanol gradient tube after 18 h of ultracentrifugation showing that the EVs concentrate around fraction 7. (B) NanoFCM analysis of all fractions obtained from the gradient showing an enrichment of particles around fractions 7 and 8.

EV diameter size distribution as evaluated by NanoAnalyzer (Figure 3D), ranging from 50 to 200 nm with an average mean size of 70 nm, and by NTA (Figure 3E) with a distribution of diameter size ranging from 100 – 500 nm and average mean of 200 nm. Using the concentrations of iodixanol described here (Step 2), EVs remain mainly in fractions 7 and 8 of the density gradient (Figure 4). After iodixanol gradient, we typically recover 50% of EV initial preparation considering all 12 fractions, with around 75% of the recovered EVs present in the Fractions 7 and 8 of the gradient (Figure 4B).

## Limitations

### Sample heterogeneity and purity

The optimized version of the described protocols represents a great improvement over the previous one,^8^ which relied on the use of fungal culture supernatants. The advancement of the protocol designed to obtain EV from solid culture media^3^ is particularly evident in terms of both yield and time efficiency. Nevertheless, a major limitation persists namely the particle heterogeneity in the composition of the final sample. This issue stems from the fact that the protocols rely on centrifugation steps, which effectively separate various particles but concurrently concentrate diverse EV subpopulations and other carryover particles. This might be especially important for the Step 1a protocol, in which the ultracentrifugation step is bypassed. Moreover, our understanding of fungal EV subpopulations comprising decorated and non-decorated EVs in *C. neoformans*, for instance, and their biogenesis remains limited, which could potentially influence the molecules they carry and hinder the discovery of appropriate markers. Therefore, additional research is imperative to address these knowledge gaps comprehensively.

In the conditions when purity is a priority, (e.g., to perform omics analysis) possible solutions include the employment of EV size exclusion chromatography (SEC) columns, combined with density gradient enrichment (Step 2), followed by an in-depth analysis of the sample by electron microscopy techniques to visualize in which fraction the EVs are concentrated. It is important to acknowledge that there remains the possibility that non-EV particles with similar density cluster in the same EV fraction. However, the quantity of these impurities obtained by density gradients is expected to be lower compared to that obtained through standard protocols of direct ultracentrifugation without a gradient step. Recent studies in *C. neoformans* using Iodixanol gradient revealed that RNAs are co-sedimented with EVs, especially in fractions 6,7 and 8.^10^ For *C. deuterogattii*, the highest particle number was also detected in fraction 7, along with the highest concentration of the major cryptococcal polysaccharide, glucuronoxylomannan.^4^ It is worth noting that this approach does have drawbacks, notably the increased time consumption and reduced yield that should be considered case by case.

### Lack of fungal EV markers

In contrast to mammalian EVs, there is a lack of consensus and standardization regarding the potential markers for fungal EVs which could greatly facilitate the isolation and characterization steps, even allowing their study during fungal infections of different *in vivo* models. The identification of abundant EV-associated proteins such as the mannoprotein mp88 in *C. neoformans* and other protein families, such as the tetraspanins associated with fungal EV membranes,^1^^,^^11^ could represent good candidates. However, a consensus has not yet been reached, as evidence suggest potential variations among fungal species.

### Characterization of particles based on size and sterol content

Another point of attention is regarding the single-EV characterization procedures since all methodologies available so far present technical limitations. For the ones described here, it is known that the nanoFCM is unable to detect particles smaller than 40 nm. As for Nanosight, even being able to detect particles from 10 nm (according to the manufacturer), the results obtained usually represent particles larger than 100 nm, which reflects on considerable differences in EV median diameter size among the two apparatus. The limitations of size detection might also affect an accurate concentration measurement. Due to these limitations, assessing the single EV morphology by electron microscopy techniques is essential when describing for the first time EVs released by a particular strain and/or species.

In relation to Amplex Red Cholesterol Assay, it is an indirect way of indicating the amount of EVs in the sample based on ergosterol content. This assay can be highly useful especially when proceeding with EV production screenings (Step 1a). However, there are limitations once the technique quantifies total sterols of any origin, EV- related or not. That said, an effective approach would be combine two different methodologies that yield distinct outputs to confirm the EV content of a particular fungal strain or experimental condition.

### Storage

According to the MISEV2023 guidelines, it is crucial to consider how pre-separation storage can affect the eventual separation of EVs. Additionally, it is important to either reduce the frequency of freeze-thaw cycles or assess the effects of these cycles on EV preparations.^5^ Based on our experience, we recommend processing the cell suspension promptly after it is collected from the solid medium. Additionally, we advise storing EV preparations, diluted in 0.22 μm-filtered PBS 1x and aliquoted, at −80°C. However, it is essential to approach this method cautiously, considering individual cases on a case-by-case basis.

## Troubleshooting

### Problem 1

When comparing the EV production for different strains or experimental conditions is possible to have different growth curves on selected culture media. In *C. neoformans*, as the EV production is related to the growth curve,^2^ this is a problem that should be considered, especially regarding the EV isolation for the screening of mutant collections (Step 1a).

### Potential solution

In such cases, a different time point to collect the cells and obtain the EVs should be considered, according to the optimal time point provided by the detailed growth curve analyses.

### Problem 2

A low yield of particles following Steps 1a, 1b and/or Step 2.

### Potential solution

It is possible to collect cells from several plates until a good particle yield is reached. However, we suggest using the proportion 1 plate per 1 mL of PBS 1x (minimum). Alternatively, it is also possible to pool samples to acquire an adequate amount of EVs for various research purposes. In accordance with the MISEV 2023 guidelines,^5^ if it becomes necessary to pool samples, it is important to note the number of individual samples included in the pool, the amount of cells contributing to the pool, the volume of each individual sample, and the final quantity. Whenever feasible, it is important to provide additional details about the individual samples involved.

### Problem 3

Despite the double centrifugation of cells collected from the agar plates in Step 1a and Step 1b, the supernatant can still contain cells depending on the cell polysaccharide capsule size and supernatant viscosity. The presence of cells could be noticed, for instance, by any turbidity in the supernatant.

### Potential solution

In such cases, it is recommended to add an additional centrifugation step to remove the remaining cells. Centrifuge the sample according to the last speed level for each protocol (either Step 1a or 1b) and transfer the supernatant to a new tube without touching the pellet. To verify the absence of cells in the EV preparation, an aliquot can be plated on a rich medium, such as a YPD agar plate, and incubated for 48 h at 30°C. The absence of growth will confirm sterility.

## Resource availability

### Lead contact

Further information and requests for resources and reagents should be directed to and will be fulfilled by the lead contact, Juliana Rizzo (juliana.rizzo@biof.ufrj.br).

### Technical contact

Questions about the technical specifics of performing the protocol should be directed to and will be answered by the technical contact, Alicia C. Piffer (alicia.corbellini-piffer@pasteur.fr).

### Materials availability

This study did not generate new unique reagents.

### Data and code availability

The published article includes a compilation of both datasets generated and analyzed during this study and the datasets obtained in the paper available at Rizzo et al., 2023.^2^ This study did not generate or analyze any code.
